# Active site loops of membrane-anchored metallo-β-lactamases from environmental bacteria determine cephalosporinase activity

**DOI:** 10.1128/aac.01918-24

**Published:** 2025-06-23

**Authors:** Matías C. Carnevale, Antonela R. Palacios, Philip Hinchliffe, Juliana Delmonti, Salvador I. Drusin, Diego M. Moreno, Robert A. Bonomo, James Spencer, Alejandro J. Vila

**Affiliations:** 1 Laboratorio de Metaloproteínas, Instituto de Biologia Molecular y Celular de Rosario (IBR, CONICET-UNR) 63031, Rosario, Argentina; 2 School of Cellular and Molecular Medicine, University of Bristol 1980, Bristol, United Kingdom; 3 Instituto de Quimica de Rosario (IQUIR, CONICET-UNR) 370496 , Rosario, Argentina; 4 Facultad de Ciencias Bioquímicas y Farmacéuticas, Universidad Nacional de Rosario 28237, Rosario, Argentina; 5 Department of Biochemistry, Case Western Reserve University School of Medicine 12304, Cleveland, Ohio, USA; 6 Department of Medicine, Case Western Reserve University School of Medicine 12304, Cleveland, Ohio, USA; 7 Louis Stokes Cleveland Department of Veterans Affairs Medical Center 20083, Cleveland, Ohio, USA; 8 Department of Molecular Biology and Microbiology, Case Western Reserve University School of Medicine 12304, Cleveland, Ohio, USA; 9 CWRU-Cleveland VAMC Center for Antimicrobial Resistance and Epidemiology, Cleveland, Ohio, USA; 10 Department of Pharmacology, Case Western Reserve University School of Medicine 12304, Cleveland, Ohio, USA; 11 Department of Proteomics and Bioinformatics, Case Western Reserve University School of Medicine 12304, Cleveland, Ohio, USA; University of Fribourg, Fribourg, Switzerland

**Keywords:** metallo-beta-lactamase, NDM, *Chryseobacterium* spp., membrane-anchoring

## Abstract

Antimicrobial resistance is a significant global public health threat that limits treatment options for bacterial infections. This situation is aggravated by the environmental spread of β-lactamase genes. In particular, metallo-β-lactamases (MBLs) hydrolyze almost all available β-lactam antibiotics, including late-generation cephalosporins and carbapenems. Among MBLs, the New Delhi metallo-β-lactamase (NDM-1) of subclass B1 has shown the most ominous dissemination. NDM variants are the only MBLs of clinical importance that are membrane-anchored, a sub-cellular localization that endows them with high stability under conditions of metal limitation. However, antibiotic resistance predates modern antibiotic usage, and environmental bacteria serve as reservoirs for resistance genes. Here, we report the biochemical and structural characterization of two membrane-bound MBLs: CJO-1 and CIM-2, from *Chryseobacterium joostei* and *Chryseobacterium indologenes*, respectively. Both MBLs confer β-lactam resistance on producer bacterial strains and hydrolyze several antibiotics, although with impaired efficiency compared to NDM-1. Crystal structures reveal differences, compared to previously studied B1 MBLs, in the active site loops and their dynamic properties that impact activity. Specifically, a hindered access to the active site with the contribution of a Tyr residue in loop L10 and the presence of a positively charged Lys residue in loop L3 limit hydrolysis of cephalosporins with charged C3 substituents. Some of these novel features are preserved in other MBLs from *Chryseobacterium* spp. These findings suggest that *Chryseobacterium* spp. could act as reservoirs of MBL genes, while informing on the diversity of structure-function relationships and dynamic behaviors within the B1 subclass of these enzymes.

## INTRODUCTION

The global increase in and dissemination of antimicrobial resistance (AMR) is one of the main global public health threats and is forecast to become the attributable cause of death of almost 2 million people and to be associated with more than 8 million deaths by 2050 (1). The emergence and spread of drug-resistant pathogens that have acquired new resistance mechanisms continue to threaten our ability to treat common infections (2). One of the main drivers of resistance is the selective pressure generated by the misuse and/or abuse of antibiotics. In its latest report, the World Health Organization has identified 24 pathogens in the bacterial priority list of pathogens that span 15 families of antibiotic-resistant species (3). The ESKAPE group (*Enterococcus faecium*, *Staphylococcus aureus*, *Klebsiella pneumoniae*, *Acinetobacter baumannii*, *Pseudomonas aeruginosa,* and *Enterobacter* spp.) was identified as key resistant pathogens of high clinical importance, particularly with respect to healthcare-associated infections. In gram-negative group members (*K. pneumoniae*, *A. baumannii*, *P. aeruginosa,* and *Enterobacter*/Enterobacterales) and other gram-negative species of high and critical priority, the main mechanism of resistance to β-lactam antibiotics (the largest group of antimicrobial drugs) is the production of β-lactamases (4, 5).

β-Lactamases can be divided into four classes (A, B, C, and D), based on their sequence homology (5). The main group of carbapenemases (i.e., enzymes challenging carbapenems, the most potent β-lactams) corresponds to the class B or metallo-β-lactamases (MBLs), whose activity depends on the presence of one or two zinc ions in their active site (6). These enzymes display a broad substrate spectrum, being capable of hydrolyzing penicillins, cephalosporins, and carbapenems. The expression of MBLs in gram-negative pathogens is usually accompanied by the expression of other resistance determinants, giving rise to difficult-to-treat organisms. Despite recent progress, clinically approved MBL inhibitors are yet to be commercially available (7, 8). MBLs show relatively low sequence identity (<20%) between enzymes from their three subclasses (B1, B2, and B3). The enzymes of subclass B1 are those with the greatest clinical impact (9), including the plasmid-encoded enzymes, IMP-1, VIM-2, and NDM-1 and -5.

The NDM (New Delhi metallo-β-lactamase) family has shown the fastest and largest geographical spread observed to date for an MBL, involving more than 80 countries (10). Surprisingly, this phenomenon is not related to a specific clone, plasmid, or a single type of genetic structure (11), but is related to specific features of this protein family (12). NDM variants present a broad substrate range, similar to most B1 enzymes, but are unique among MBLs in being membrane-anchored enzymes, while other known B1 β-lactamases are soluble, periplasmic proteins (13, 14). This sub-cellular localization is due to the presence of a lipidation sequence, called a lipobox, in the signal peptides of all NDM variants (14). After the precursor is exported from the cytoplasm, the signal peptide is cleaved, and a diacylglycerol group is transferred to the free sulfhydryl of the lipobox cysteine, which is the only residue of the lipobox sequence retained in the mature protein. Membrane anchoring was reported to stabilize NDM-1 under conditions of zinc starvation, as well as to favor secretion of NDM-1 in outer membrane vesicles, i.e., disseminating the protein beyond the cellular boundaries (14). Overall, this sub-cellular localization provides an evolutionary advantage to NDM-producing bacteria (15).

NDM-1 and its 79 allelic variants known to date (http://www.bldb.eu) (16) are the only membrane-anchored MBLs of clinical impact. This raises the question of the evolutionary origin of NDM enzymes as lipoproteins. The first six residues of the NDM signal peptide sequence are identical to those of the AphA6 aminoglycoside phosphotransferase from *Acinetobacter baumannii* (17). However, the evolutionary origin of its lipidation sequence is unknown.

Until 2024, NDM variants represented the only group of MBLs experimentally characterized as lipoproteins. Recently, the presence of a B1 MBL from a resistant *Chryseobacterium indologenes* isolate was reported, with the enzyme named CIM-1 (*C. indologenes* MBL) (18). CIM-1 possesses a lipobox in the signal peptide and has been experimentally characterized as a membrane-bound enzyme. The Beta-lactamase database (16) describes 29 β-lactamases from *Chryseobacterium* spp., of which 23 have been found in *C. indologenes* (CIM-1, CIA variants, and almost all IND variants), 2 in *C. gleum* (CGA-1 and CGB-1), 1 in *C. piscium* (CPS-1), 1 in *C. mole* (CMQ-1) as chromosomally encoded genes, and 2 from genomic libraries (CHM-1 and IND-17). Five of the 29 identified β-lactamases from *Chryseobacterium* spp. are extended-spectrum class A β-lactamases (CGA-1 and four variants of CIA). The rest belong to class B, 2 of them, CPS-1 and CMQ-1, being B3 MBLs, and 22 B1 enzymes (CGB-1, CHM-1, CIM-1, and 19 variants of IND-1).

The genus *Chryseobacterium* of the *Flavobacteriaceae* family includes gram-negative bacteria widely distributed in natural environments, such as water, soils, rhizospheres, plants, frogs, chicken, fish, and raw milk (19, 20). Members of the *Chryseobacterium* genus are intrinsically multidrug-resistant organisms that evade both carbapenems and colistin (21). *Chryseobacterium* may cause various clinical syndromes that are not always responsive to standard therapies, due mainly to this intrinsic resistance to several antimicrobial classes. *C. indologenes* is the most commonly isolated *Flavobacteriaceae* from clinical specimens and is associated with different types of infections, such as intra-abdominal and urinary tract infections, catheter-related bacteremia, cellulitis, sepsis, and pneumonia (22–24). In a retrospective study of 215 clinical isolates of *C. indologenes* in Taiwan, the most prevalent co-infection pathogen in patients with *C. indologenes* pneumonia was *Acinetobacter baumannii* (39.6%), followed by *Pseudomonas aeruginosa* (25.3%) and *Klebsiella pneumoniae* (14.3%) (25).

Here, we report an analysis of the ubiquity of subclass B1 metallo-β-lactamases containing lipoboxes in different *Chryseobacterium* spp. In addition to CIM-1, we identified an additional 70 B1 MBLs containing lipoboxes, including the previously reported CHM-1 (26). We also report the biochemical and structural characterization of two of these enzymes: an MBL from *C. indologenes*, CIM-2, and another from *Chryseobacterium joostei*, CJO-1. Both are membrane-anchored enzymes and able to protect recombinant producers from a broad spectrum of β-lactam antibiotics; however, they displayed impaired activity toward some substrates, compared to other B1 MBLs. We propose that the selectivity against some cephalosporins can be explained by the presence of a positively charged residue in the flexible active site loop L3 and hindered access to the active site. Overall, these results suggest that *Chryseobacterium* could act as an environmental reservoir of membrane-bound MBLs.

## RESULTS

### Bioinformatics search of lipidated MBL sequences in *Chryseobacterium* spp.

With the aim of examining the ubiquity of membrane-bound metallo-β-lactamases in *Chryseobacterium*, we searched for *Chryseobacterium* MBLs in the NCBI non-redundant protein database to find subclass B1 MBLs using NDM-1 as a target sequence. We filtered the search using the Signal P 6.0 server (27), looking for those sequences containing the consensus ([LVI][ASTVI][GAS][C]) or an alternative non-canonical lipobox sequence in their N-terminal signal peptide (see below). We then used a more selective filter, upholding those sequences which also displayed the characteristic ligand set from B1 MBLs, characterized by the HXHXD and GGC sequences. This search resulted in the finding of 71 protein sequences, which include the recently reported CIM-1 (18) and CHM-1 (26). The remaining 69 proteins are not listed in the Beta-lactamase database (16) (Fig. S1). Within this group, two putative MBLs were identified as chromosomally encoded proteins in *Chryseobacterium* spp., which we named CIM-2 and CJO-1. CIM-2, like the MBL CIM-1, was found in *C. indologenes*, an opportunistic pathogen of growing importance in the clinic which can express 19 variants of the soluble B1 MBL, IND-1, while CJO-1 was identified as a putative MBL sequence from the environmental organism *C. joostei*, first isolated in South Africa from dairy tanks.

### Comparative sequence analysis of CIM-2 and CJO-1

Previous work has reported the presence of lipoboxes in all NDM alleles and in MBLs from all three subclasses, B1, B2, and B3, resulting in membrane-bound proteins (14). This sub-cellular localization favors protein secretion into outer membrane vesicles and protects the protein from degradation under conditions of Zn(II) starvation (14).

**Fig 1:**
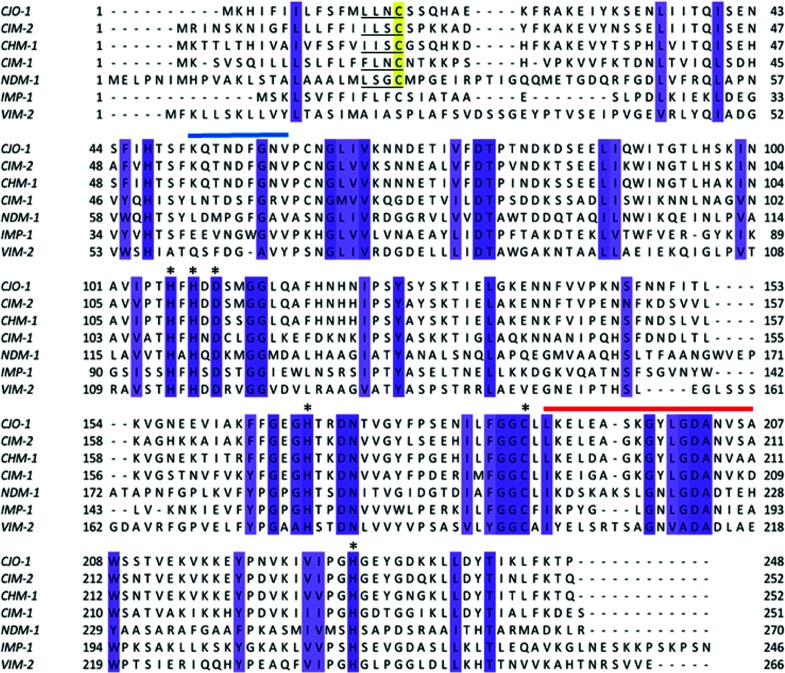
Protein sequence alignment of CJO-1 and CIM-2 with NDM-1, IMP-1, VIM-2, and other *Chryseobacterium* B1 MBLs. The consensus Zn(II) ligands are indicated with an asterisk (*), putative lipobox sequences are underlined, and lipoboxes Cys are highlighted in yellow. The numbers indicate the residue positions at the ends of the sequences which differ from the standard numbering (BBL numbering [28, 29]). Loop L3 is indicated by a horizontal blue bar and corresponds to residues 59–67 in the standard numbering; loop L10 is indicated by a horizontal red bar and corresponds to residues 223–241 in the standard numbering. Dashes indicate gaps to align the sequences. The sequence alignment was visualized with Jalview (30).

Sequence analysis of CJO-1 and CIM-2 using Signal P 6.0 revealed putative lipobox sequences at their N-termini signal peptides (Fig. 1, underlined). The sequences found for CJO-1 and CIM-2 were LLNC and ILSC, where the cysteine (C) residue is present at positions 16 and 20, respectively. Both sequences differ from the consensus lipobox sequence found in NDM-1, LSGC, where the cysteine is present at position 26 (14).

Both CJO-1 and CIM-2 present the characteristic ligand set corresponding to the conserved Zn(II) binding motif in B1 MBLs: residues His116-His118-His196 and Asp120-Cys221-His263, respectively, according to the BBL numbering (28, 29). Pairwise alignments reveal that CJO-1 and NDM-1 share a 25.1% sequence identity, while CIM-2 showed a 23.9% sequence identity with NDM-1 and 74.6% with CJO-1. Both putative MBLs showed homology with CHM-1: 76.6 and 77.4% sequence identity, respectively. CHM-1, identified from a *Chryseobacterium* spp. genomic library, also possesses a lipobox in its signal peptide. The recently described CIM-1 exhibits 52.5% and 50.8% sequence identity with CJO-1 and CIM-2, respectively. In comparison with other *Chryseobacterium* MBL B1 enzymes (CGB-1, IND-1) and VIM-2, both CJO-1 and CIM-2 showed low percentages of sequence identity, ranging from 26.7 to 29.8%.

Comparison of the sequence of mature, processed NDM-1 with those of CJO-1 and CIM-2 reveals substitutions of several residues involved in important motifs flanking the active site, such as loop L3 (residues 59–67, highlighted by a blue bar in Fig. 1) and loop L10 (residues 223–241, highlighted by a red bar in Fig. 1). Nevertheless, the conservation of the Zn(II) ligands, and many of the residues from loops L3 and L10, suggests that CJO-1 and CIM-2 are B1 MBLs. The presence of a lipobox predicts them to be lipidated, membrane-anchored enzymes.

### CIM-2 and CJO-1 are membrane-anchored enzymes

**Fig 2:**
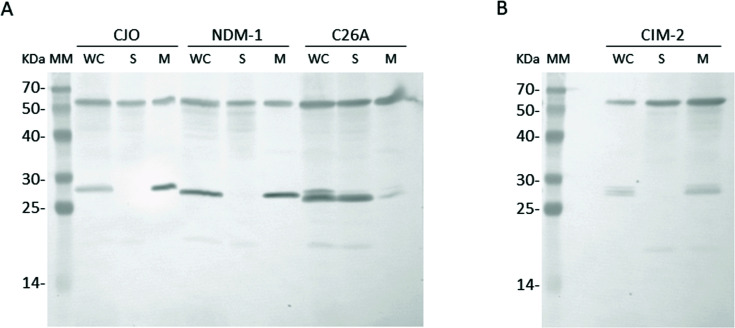
Immunodetection of the putative *Chryseobacterium* MBLs CJO-1 (**A**) and CIM-2 (**B**) expressed in *Escherichia coli*. The lipoprotein NDM-1 is included as a positive control of a membrane-bound MBL. WC, whole cells; S, soluble fraction of the lysate; M, total membranes of the lysate; MM, molecular marker. The bands between 25 and 30 kDa correspond to the expressed MBLs. The band at 60 kDa corresponds to GroEL, used as a control.

To assess the sub-cellular localization of these novel *Chryseobacterium* MBLs, we cloned the full-length *bla*_CIM-2_ and *bla*_CJO-1_ genes into the pMBLe vector. This plasmid allows the expression of MBLs with their own signal peptide and an additional StrepTag-II sequence (eight residues) fused to their carboxy-terminal end aimed to standardize immunodetection. The addition of the StrepTag-II sequence does not interfere with the β-lactamase activity and allows their immunodetection using commercial anti-StrepTag-II antibodies (14). Furthermore, pMBLe has the *pTac* promoter, which is inducible by isopropyl-β-D-1-thiogalactopyranoside (IPTG), enabling a wide range of induction levels capable of producing amounts of MBLs equivalent to those found in clinical strains. Using this expression system, we expressed both proteins and assessed their localization by lysing the cell cultures, separating the cytoplasm, periplasm, and membrane fractions by ultracentrifugation, followed by immunodetection. As shown in Fig. 2, both CJO-1 and CIM-2 were found in the membrane fraction of the lysate (M), with no traces of either protein in the soluble fraction (cytoplasm and periplasm, S). The same pattern was observed for NDM-1, while the opposite behavior was displayed by the NDM-1 C26A mutant. NDM-1 C26A lacks the cysteine residue that is the site of lipidation and is thus a soluble periplasmic, rather than membrane-anchored, enzyme and appears in the soluble fraction of the lysate. These results confirm that both CJO-1 and CIM-2 are lipidated, membrane-anchored proteins.

### CJO-1 and CIM-2 confer protection against all classes of bicyclic β-lactam antibiotics

We then tested the ability of these proteins to confer a β-lactam resistance phenotype upon a bacterial host. We employed the same expression system used to establish their sub-cellular localization and challenged *E. coli* cells expressing either CJO-1 or CIM-2 with representative antibiotics from the different β-lactam families to determine their minimum inhibitory concentrations (MICs).

The data summarized in Table 1 show that CJO-1 and CIM-2 were able to protect *E. coli*, as revealed by the increased MICs, from almost all tested β-lactams. Both proteins showed similar resistance profiles for all tested antibiotics, displaying MIC values differing by at most onefold dilution. Both *Chryseobacterium* proteins increased the MICs with respect to a strain containing the empty vector: against piperacillin by 4-fold, imipenem by 2-fold, meropenem by 8-fold and cefotaxime by 128-fold. CJO-1 and CIM-2, respectively, increased the MICs against ceftazidime by 256- and 512-fold, i.e., with the differences between the two enzymes falling within the experimental error. Strikingly, none of them showed a significant impact in the MIC of cefepime compared to the strain with the empty vector.

**TABLE 1:** MICs (µg/mL) for *E. coli* expressing CJO-1, CJO-1 K59L, CIM-2, and NDM-1

Drug	MIC (µg/mL) for *E. coli* DH5α				
	pMBLe	pMBLe::CJO-1	pMBLe::CJO-1 K59L	pMBLe::CIM-2	pMBLe::NDM-1
Penicillins					
Piperacillin	1	4	–^*a*^	4	16
Carbapenems					
Imipenem	0.25	0.5	–	0.5	2
Meropenem	0.03125	0.25	–	0.25	1
Cephalosporins					
Cefotaxime	0.0625	8	4	8	32
Cefepime	0.0625	0.0625	0.5	0.125	8
Ceftazidime	0.25	64	32	128	2,048

^*a*^ –, not tested.

Bacteria expressing NDM-1 showed further increases in MICs compared to those expressing CJO-1 and CIM-2, being able to grow in fourfold higher concentrations when tested against piperacillin, carbapenems, and cefotaxime (Table 1), and 32- and 16-fold higher concentrations when tested against ceftazidime. In the case of cefepime, NDM-1 is able to confer some resistance, in contrast to both CJO-1 and CIM-2. These results indicated that when expressed in *E. coli,* these putative *Chryseobacterium* MBLs were able to increase the MICs to almost all tested β-lactams*,* though to a lesser extent than NDM-1.

### Cephalosporinase activity of CJO-1 and CIM-2 is determined by the nature of the C3 substituent

To analyze the activity of these putative MBLs, we expressed and purified both CJO-1 and CIM-2 in their truncated, soluble versions, i.e., that lack the signal peptide. Then we determined the steady-state kinetic parameters for hydrolysis of a panel of β-lactams and compared the results with those obtained for NDM-1 (Table 2).

**TABLE 2:** Steady-state kinetic parameters for CJO-1, CIM-2, and NDM-1

Substrate	CJO-1			CIM-2			NDM-1		
	*K*_M_ (µM)	*k*_cat_ (s^−1^)	*k*_cat_/*K*_M_ (µM^−1^s^−1^)	*K*_M_ (µM)	*k_c_*_at_ (s^−1^)	*k*_cat_/*K*_M_ (µM^−1^s^−1^)	*K*_M_ (µM)	*k*_cat_ (s^−1^)	*k*_cat_/*K*_M_ (µM^−1^s^−1^)
Penicillins									
Piperacillin	1,900 ± 300	30 ± 4	0.016 ± 0.005	2,400 ± 500	48 ± 7	0.020 ± 0.007	120 ± 10^*a*^	1,190 ± 40^*a*^	10 ± 1^*a*^
Carbapenems									
Imipenem	2,700 ± 500	280 ± 40	0.10 ± 0.03	2,300 ± 300	2,000 ± 200	0.9 ± 0.2	150 ± 30^*a*^	570 ± 30^*a*^	4 ± 1^*a*^
Cephalosporins									
Cefotaxime	126 ± 4	528 ± 6	4.2 ± 0.2	180 ± 20	820 ± 30	4.7 ± 0.7	53 ± 3^*b*^	332 ± 5^*b*^	6.3 ± 0.4*^b^*
Cefepime	440 ± 60	19 ± 2	0.043 ± 0.009	700 ± 200	28 ± 5	0.04 ± 0.02	50 ± 10^*a*^	300 ± 20^*a*^	6 ± 2^*a*^
Ceftazidime	1,000 ± 400	110 ± 30	0.10 ± 0.06	2,000 ± 300	380 ± 50	0.18 ± 0.05	60 ± 10^*a*^	620 ± 20^*a*^	10 ± 2^*a*^

^*a*^ Palacios et al. (31).

^*b*^ Khan and Rehman (32).

The highest observed catalytic activity for both enzymes was against cefotaxime, with *k*_cat_/*K_M_* values in the same order of magnitude as those reported for clinically relevant B1 MBLs, such as NDM-1 (Table 2) (31, 32). Both enzymes had lower catalytic efficiencies against the other antibiotics tested. These differences were in general due to high *K_M_* values, ≥2 mM for piperacillin and imipenem, and slightly lower values (1–2 mM) for ceftazidime, suggesting impaired binding of these substrates to both enzymes compared to NDM-1. In consequence, CJO-1 and CIM-2 had catalytic efficiencies for hydrolysis of imipenem and ceftazidime one order of magnitude smaller than the respective values for NDM-1. Cefepime hydrolysis was characterized by an intermediate *K_M_* value but also a low *k*_cat_ value, making this, together with piperacillin, the poorest substrate tested for both enzymes. The activity trend for cephalosporins was cefotaxime > ceftazidime > cefepime (Table 2).

CJO-1 and CIM-2 were therefore able to hydrolyze all assayed β-lactam antibiotics, showing very similar catalytic efficiencies to one another. Their cefotaximase activity was as high as that determined for NDM-1; however, they presented lower activities against the rest of the tested β-lactams.

### CIM-2 and CJO-1 show a B1 MBL fold with changes in the active site loops

**Fig 3:**
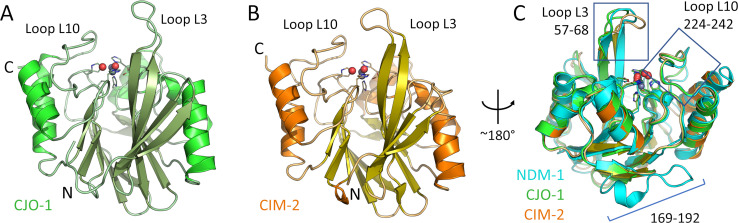
Crystal structures of CJO-1 and CIM-2. Zn coordination residues are represented by sticks, Zn ions by gray spheres, and water molecules by red spheres. X-ray structure of CJO-1 (green, PDB 9GX9) from *C. joostei* (**A**). X-ray structure of CIM-2 (orange, PDB 9GX8) from *C. indologenes* (**B**). Superimposition of CJO-1 (green), CIM-2 (orange), and NDM-1 (cyan, PDB 5ZGY), showing the positions of key active site loops and the extended NDM-1 loop at position 169–192 (**C**).

Aiming to understand the substrate profile of these enzymes, we crystallized both CJO-1 and CIM-2 in their soluble forms and solved their X-ray structures using single wavelength anomalous dispersion (SAD) from data collected at the Zn edge (Table S1). CIM-2 diffraction data extended to 1.56 Å resolution, and CJO-1 to 1.34 Å. The structures confirmed that both proteins adopt the characteristic αβ/βα sandwich fold of B1 subclass MBL enzymes (5) (0.59 Å root mean square deviation [RMSD], over 220 C_α_ of CJO-1 with CIM-2), with two Zn(II) ions in their active sites (Fig. 3A and B, respectively). CJO-1 could be modeled with a partially oxidized Cys221, as has been observed previously in crystal structures of the B1 MBL VIM-2 (33), with 13% occupancy for a sulfenic acid (34). The overall folds of CJO-1 and CIM-2 are therefore similar to that of NDM-1, with RMSDs (calculated using PDBeFold) of 1.33 Å (CJO-1, over 212 C_α_ residues) and 1.35 Å (CIM-2, 214 C_α_) against NDM-1 (PDB 5ZGY). The most significant structural changes, compared to NDM-1, are observed over the largely non-conserved residues 169–192, in which there is a characteristic four-residue insertion of NDM-1 and its variants (16) among B1 MBLs (Fig. 3C).

CJO-1 and CIM-2 have the same conserved active site residues coordinating Zn1 (His116, His118 and His196) and Zn2 (Asp120, Cys221 and His263), and so have highly similar active site architectures, with similarity extending to the positioning of their active site water molecules (Fig. S2A; Table S2). However, compared to NDM-1, we note there are minor differences with respect to the conformation of the His118 side chain that coordinates Zn1, as well as a displacement, after superimposition, of the apical Zn2 coordinated water (Wat2) by 1.2 Å from its position in NDM-1 (Fig. S2B; Table S2).

**Fig 4:**
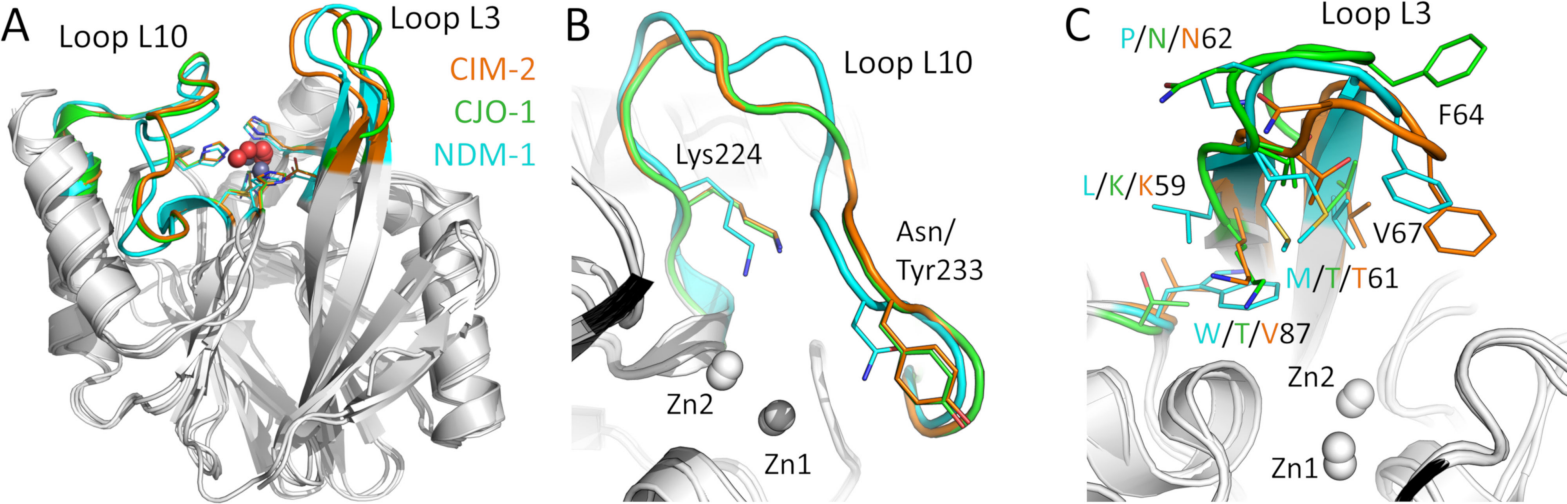
Active site loops in CJO-1, CIM-2, and NDM-1. CJO-1 is colored green, CIM-2 orange, and NDM-1 cyan. Zn coordination residues and significant substitutions are presented as sticks; Zn ions (Zn1 and Zn2) by gray spheres and water molecules by red spheres. Superimpositions of CJO-1, CIM-2, and NDM-1, with loops L3 and L10 colored according to the protein (**A**). Loop L10 conformations in CJO-1, CIM-2, and NDM-1, with active site residues involved in ligand binding highlighted (**B**). Loop L3 conformations in CJO-1, CIM-2, and NDM-1, with significant amino acids around the active site labeled (note, including at position 87 which is not on loop L3, but is potentially involved in ligand binding) and colored according to their respective proteins (**C**).

Loops L10 and L3 flank the shallow groove that encloses the active site of B1 MBLs, including CJO-1, CIM-2, and NDM-1 (Fig. 4A)**,** and have almost identical sequences in CJO-1 and CIM-2 (Fig. 1). Loop L10 adopts similar conformations in CJO-1 and CIM-2, which are distinct from its conformation in the NDM-1 crystal structure (Fig. 4B), likely due to the presence of an extra amino acid in this loop in NDM-1, and further differences in amino acid sequence (Fig. 1). Indeed, Asn233, present in NDM-1 and in most B1 MBLs, and previously proposed to interact with the carbonyl group of the β-lactam substrate (35–39), is substituted by a Tyr in CJO-1 and CIM-2, as observed in some other B1 MBLs such as BlaB (40) and SPM-1 (34), and in all characterized (16) B1 MBLs from *Chryseobacterium* spp. (Fig. 1). However, the position of Lys224 is conserved in CJO-1, CIM-2, and NDM-1, enabling potential interactions with the carboxylate group of the β-lactam, which are observed in structures of NDM-1 in complex with hydrolyzed substrates (41, 42).

In B1 MBLs, loop L3 can operate as a flexible flap whose residue composition and conformational changes are involved in modulating substrate accessibility to the active site and positioning of β-lactam molecules bound to the metal site (31, 43, 44). Reflecting this flexibility, overall conformations of L3 differ between NDM-1 and CJO-1/CIM-2 (Fig. 4C), and between CJO-1 and CIM-2, despite their high sequence identity in this region. A conserved hydrophobic residue, Phe64, is present at the tip of loop L3 in CJO-1, CIM-2, and NDM-1. However, Met61 and Trp87, which are part of a hydrophobic patch in NDM-1 and have been observed to be important for binding some inhibitors (36, 37), are not conserved in CJO-1 (Thr61 and Thr87) or CIM-2 (Thr61 and Val 87).

### Residues in loops L3 and L10 define substrate specificity

**Fig 5:**
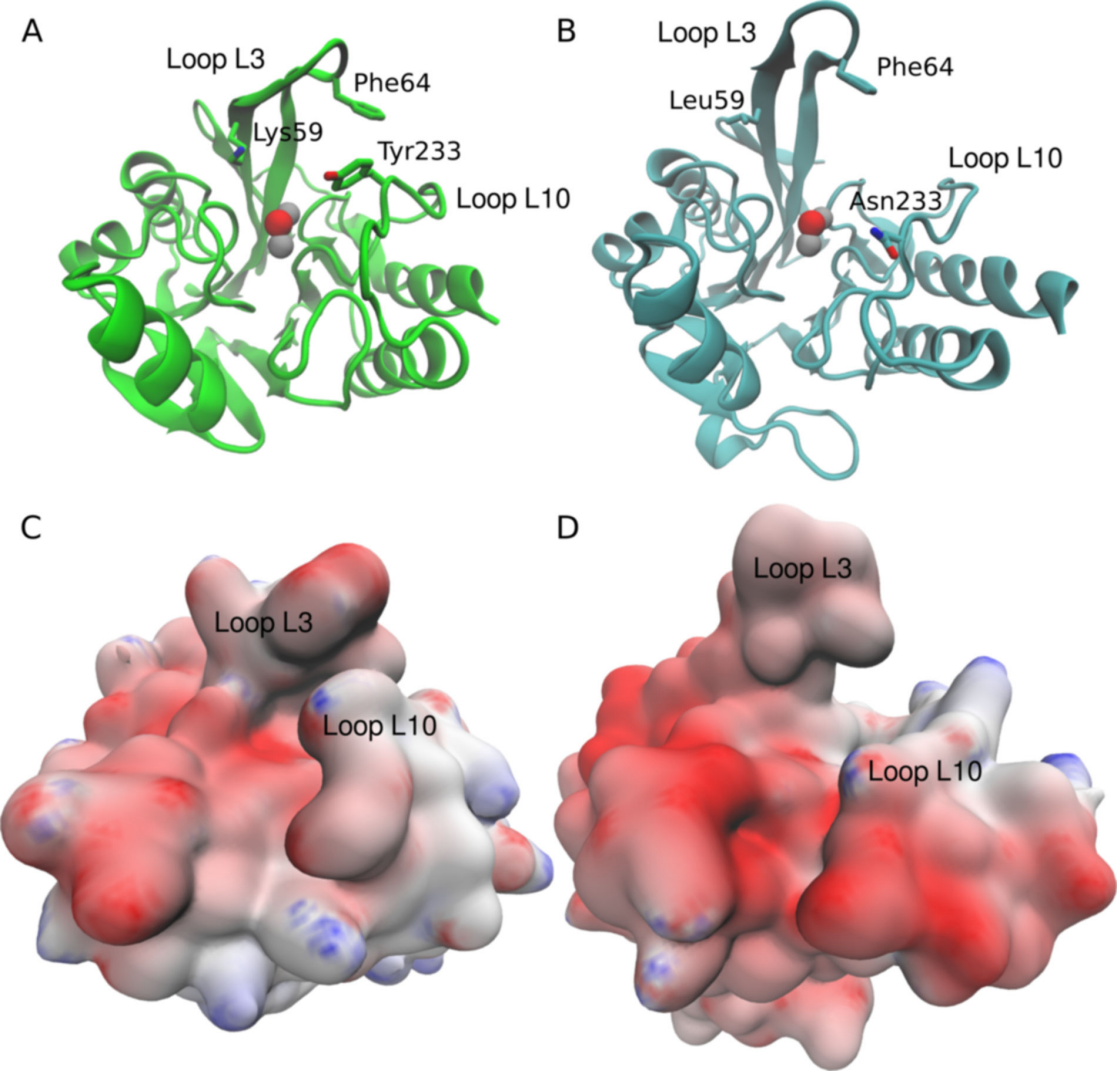
Representative structures obtained from molecular dynamics simulations. CJO-1 (**A**) and NDM-1 (**B**) are shown as cartoon representations with the catalytic Zn(II) ions and the oxygen atom of the catalytic hydroxyl ion shown as gray and red spheres, respectively. The electrostatic potential surfaces of CJO-1 (**C**) and NDM-1 (**D**) with negative, neutral, and positive potentials colored as red, white, and blue, respectively, in a scale from −8 to 8 *k*_B_T/e_c_.

These crystal structures reveal differences, compared to previously characterized B1 MBLs, in loops L3 and L10 flanking the active sites of these *Chryseobacterium* enzymes. We therefore further explored these differences by running 1 μs of molecular dynamics simulations of the uncomplexed enzymes, based on the crystal structures of CJO-1 and NDM-1. These calculations revealed differing behaviors of the active sites, particularly in loops L3 and L10. In the simulations of CJO-1, both loops remain closer to the active site (Movie S1), resulting in hindered access for β-lactams with bulky substituents, like piperacillin (Fig. 5A and C), which showed the lowest catalytic efficiency among the tested β-lactams. In the case of NDM-1, loop L3 remains in an open position, similar to the one in the crystallographic structure, while loop L10 moves slightly away. Overall, this results in a more accessible active site for substrates (Movie S2).

Loop L3 in CJO-1 differs from the equivalent loop in NDM-1 in 8 of its 12 residues. Among these substitutions, the presence of a lysine at position 59 (with a positively charged side chain) also modifies the surface charge distribution of CJO-1 when compared to NDM-1 (Fig. 5C and D). This surface charge distribution is likely to generate repulsive interactions with substrates with a positive charge density in their substituents, as in the bicyclic β-lactams tested with positive charges in their C2/C3 substituents, e.g., cefepime, ceftazidime, or imipenem.

Another substitution in CJO-1 with potential to affect substrate hydrolysis is the presence of tyrosine at position 233. This position in NDM-1 is occupied by an asparagine residue that plays a role in the stabilization of the carboxylic acid formed during the reaction mechanism (35–39). The substitution at position 233 of CJO-1 can then imply a destabilization of the hydrolysis intermediate and product due to loss of this interaction. Additionally, the mobility of this residue, permitting intermittent interactions with Phe64, may have an effect on the entry and correct binding of the substrates in the catalytic site. Overall, the combined effects of Leu59Lys and Asn233Tyr substitutions in CJO-1 introduce a steric-electrostatic effect on the surface of the protein surrounding the active site. These changes, along with altered mobility of loops L3 and L10, may influence substrate access and interactions when compared with the accessibility and interactions described for the NDM-1 active site (Fig. 5C and D).

We decided to explore the impact of replacing Lys59 with a non-polar residue to test the electrostatic hypothesis. We generated the Lys59Leu variant of CJO-1, and we determined the resistance phenotype of *E. coli* cells expressing this variant. As summarized in Table 1, this replacement increased the MIC of cefepime, the poorest substrate of CJO-1, by almost one order of magnitude, with reduced impact on the MIC values of cefotaxime and ceftazidime. Analysis of the electrostatic surface of Lys59Leu CJO-1 reveals a distinct change, characterized by a negative region that more closely resembles that of NDM-1 (Fig. S3). Despite other features that can fine-tune the reactivity, we conclude that the main determinant of the distinct substrate profile of CJO-1 against cefepime is based on the electrostatic repulsion with the positive charge of the lys59 residue.

## DISCUSSION

AMR is one of the main threats to human health, due to the dissemination of genes coding for resistance elements, such as β-lactamases, among pathogenic bacteria. However, antibiotic resistance is ancient, and environmental resistance for β-lactams predates the discovery of penicillin in 1928 by Alexander Fleming. Environmental bacteria act as a reservoir for antibiotic resistance, while some species can interact with the microbiota of humans, animals, and/or plants, and so can be considered as “shuttle species,” fostering antibiotic resistance gene flow among bacteria (45).

Here, we report that environmental *Chryseobacterium* spp. harbor large numbers of genes encoding membrane-bound B1 MBLs, i.e., homologous to the widely disseminated NDM family. Specifically, we report the sub-cellular localization of two novel B1 MBLs, named CJO-1 and CIM-2, from *C. joostei* and *C. indologenes,* respectively. Both are lipidated and membrane-anchored MBLs. The resistance profiles of recombinant producer strains and the kinetic parameters of the purified enzymes against a series of β-lactam substrates allow us to annotate them as *bona fide* MBLs. We have also identified several distinctive features of these two MBLs that provide general information on structure-function relationships within the B1 subclass enzymes.

Both *Chryseobacterium* MBLs were able to confer protection against almost all of the tested β-lactams when expressed in *E. coli* cells, although to a lesser extent than NDM-1. Similarly, steady-state kinetics indicate that, compared to NDM-1, the two *Chryseobacterium* MBLs more poorly turn over the tested β-lactams, the exception being cefotaxime, for which *k*_cat_ values are higher compared to NDM-1 (31), although catalytic efficiencies are similar due to higher *K*_M_ values in CJO-1 and CIM-2. In the case of imipenem, CIM-2 displays a higher *k*_cat_ than NDM-1, while it is smaller for CJO-1. In both enzymes, the large *K*_M_ values also result in lower catalytic efficiencies.

The crystal structures of both enzymes show an overall conserved MBL fold; but, as seen in molecular dynamics simulations, residue differences in their active sites affect the conformations of loop L3 and surface charge distributions of the enzymes. Comparing the structural features of cephalosporin substrates with their susceptibilities to hydrolysis by CJO-1 and CIM-2, we notice a clear pattern based on the charge on their C3 substituents. Cefotaxime, which has a neutral substituent at the C3 position, is a very good substrate for both MBLs, whereas for cefepime, which bears a C3 substituent with the highest positive charge, both CJO-1 and CIM-2 showed the lowest catalytic efficiency and a smaller protective effect upon the MIC of producer bacteria. As indicated by this trend, we propose that the positively charged Lys59 impairs the hydrolysis of cefepime, ceftazidime, and imipenem. This effect is further supported by the restricted access to the active site in these simulations which, consequently, hampers the hydrolysis of piperacillin.

Based on these results, we looked for the presence of positively charged residues in loop L3 of other B1 MBLs. None of the 79 NDM variants exhibited positively charged residues in loop L3, accounting for the more efficient catalytic profile against different cephalosporin substrates. In contrast, several enzymes from the VIM (46–48)and IMP families (49–51) contain positively charged residues in this loop. For example, VIM-7 harbors a Ser60Lys substitution in loop L3 (48) with respect to VIM-2. This residue points out from the active site cavity, increasing the positive charge on the protein surface, therefore impairing hydrolysis of positively charged substrates (52). In addition, the structure of VIM-7 displays a more open L3 loop compared to that of VIM-2, a fact that has been attributed to a Pro68Ser mutation (48).

The finding of a positively charged Lys residue in loop L3 in CJO-1 and CIM-2, linked to a restricted active site, shows a concerted action of these two features that limits hydrolysis of cephalosporins with positively charged C3 substituents, particularly cefepime. The study of the Lys59Leu variant of CJO-1 confirms this hypothesis. Positively charged residues are rather ubiquitous in MBLs from *Chryseobacterium* spp. The putative lipidated CHM-1 showed a high activity against cefuroxime, a cephalosporin with a neutral C3 substituent, and a low activity against ceftazidime, in line with the presence of residue Lys59 in an equivalent position to Lys59 of CJO-1 and CIM-2 (26). A similar scenario is found for the phylogenetically distant *Chryseobacterium* IND family and CGB-1 enzymes. These enzymes conserve the positively charged Lys58 in loop L3 and, consistent with our hypothesis, show a comparable pattern of cephalosporinase activity to that observed here (53, 54). In contrast, CIM-1 lacks a positively charged residue in this position, resulting in a hydrolysis profile for ceftazidime and cefepime (18) similar to those described for NDM variants.

These findings suggest that *Chryseobacterium* spp. could act as environmental reservoirs of MBL genes, specifically membrane-bound NDM-1 homologs, capable of conferring similar levels of resistance to Enterobacterales. The two *Chryseobacterium* MBLs also reveal several distinctive features that provide general information on the structure-function relationship of the B1 subclass. Moreover, we also note that the substrate specificity may have significant implications for the novel cephalosporins that are being introduced into the clinic. Based upon data obtained herein, combinations like cefepime/taniborbactam and cefepime/zidebactam may offer unique opportunities for the treatment of infections caused by *Chryseobacterium*. Further studies are required to fully explore these potential treatment options for infections caused by this opportunistic pathogen in immunocompromised hosts, as well as to assess their potential for horizontal gene transfer.

## MATERIALS AND METHODS

### Bioinformatic search of lipobox-containing *Chryseobacterium* metallo-β-lactamases

Putative *Chryseobacterium* MBLs of the subclass B1 were obtained from the NCBI non-redundant protein database. The sequences were filtered, retaining those with a length below 350 residues and the distinctive His-X-His-X-Asp and Gly-Gly-Cys B1 MBL motifs. Afterward, sequences were analyzed with SignalP 6.0 (https://services.healthtech.dtu.dk/services/SignalP-6.0/) which uses a machine learning model to predict the presence of lipobox sequences within the putative *Chryseobacterium* MBLs signal peptides. Resulting sequences were aligned, and phylogenetic trees were constructed using the maximum likelihood method and tested with the bootstrap method (500 replicates) with MEGA (Molecular evolutionary genetic analysis) (55).

### Bacterial strains and growth conditions

*Escherichia coli* DH5α was used for expression of pMBLe::MBLs in microbiological (sub-cellular localization and MIC determinations) studies. A set of isogenic clones expressing different MBL genes (*bla*_NDM C26A_, *bla*_NDM-1_, *bla*_CJO-1,_*bla*_CJO-1 Lys59Leu_, and *bla*_CIM-2_) was constructed. Cells were routinely grown aerobically with shaking (220 rpm) at 37°C in lysogeny broth (LB) or on LB agar plates, except where indicated. Gentamicin was used where necessary at 20 µg mL^−1^ to select for the pMBLe plasmid. All reagents and chemicals were purchased from Sigma-Aldrich, except the LB culture media that were obtained from BD Difco and oligonucleotides and enzymes that were sourced from Life Technologies.

### Cloning and plasmid construction

Plasmid isolation, DNA purification, restriction enzyme digestion, ligation, and transformation were performed by standard methods according to Sambrook et al. (56). In microbiological studies (sub-cellular localization and MIC determinations), *E. coli* DH5α pMBLe::MBLs were used. CJO-1, CJO-1 Lys59Leu, and CIM-2 were synthesized by Gene Universal Inc. from their NCBI database sequences (accession number WP_076355270.1 for CJO-1 and CJO-1 Lys59Leu, and WP_062697499.1 for CIM-2) and cloned into the pMBLe vector into *Nde*I and *Hind*III sites, under the control of a β-IPTG-inducible pTac promoter. These full-length *bla*_CJO-1_, *bla*_CJO-1 Lys59Leu_, and *bla*_CIM-2_ genes were expressed with their corresponding native signal peptides and a StrepTag-II sequence fused to their C-termini. The construction of pMBLe::*bla*_NDM C26A_ and pMBLe::*bla*_NDM-1_ has been described previously (14).

For protein purification, the soluble domains, i.e., the truncated soluble versions of CJO-1 (residues 17 to 248) and CIM-2 (residues 21 to 252) were obtained through PCR amplification. Briefly, the soluble version of *bla*_CJO-1_ lacking the native signal peptide and without a C-terminal StrepTag-II sequence was PCR-amplified from pMBLe::*bla*_CJO-1_ using primers CJO-1solNdeIFw (5ʹ-GCTGCATATGAGTAGCCAGCATGCCGAA −3′) and CJO-1solRv (5′- CGGGATCCTCACGGGGTTTTAAACAGCTT-3′) and subcloned into a modified version of the pET-28a expression vector. The same procedure was applied to express *bla*_CIM-2_ using primers CIM-2solNdeIFw (5ʹ- TCTGCATATGAGCCCGAAAAAAGCCGATT −3′) and CIM-2solRv (5′- CGGGATCCTCACTGGGTTTTAAACAGATT −3′). This modified version of the pET28a plasmid (pET28a-TeV) was kindly provided by Dr. Rodolfo M. Rasia (IBR, CONICET-UNR) in which the thrombin cleavage site is replaced by a TEV protease cleavage site.

### MIC determinations

Antibacterial susceptibility was established through MIC determinations. Piperacillin, imipenem, meropenem, cefotaxime, cefepime, and ceftazidime MIC determinations for *E. coli* DH5a cells expressing the different MBLs or carrying the empty pMBLe vector (i.e., as a control lacking any MBL gene) were carried out in LB medium following the agar macrodilution method according to the Clinical and Laboratory Standards Institute protocols (57). In all cases, metallo-β-lactamase expression was induced with 20 µM IPTG.

### MBL detection in sub-cellular localization

A 100 mL aliquot of *E. coli* DH5α pMBLe::MBLs culture (OD_600_ = 1) induced for 2 h with 20 µM IPTG with shaking (220 rpm) at 37°C was pelleted, resuspended in 10 mM 4-(2-hydroxyethyl)-1-piperazineethanesulfonic acid (HEPES), 200 mM NaCl, 1 mM phenylmethylsulfonyl fluoride (PMSF), pH 7.4, and cells disrupted by sonication. Cell debris was removed by centrifugation at 14,000 × *g* and 4°C for 20 min, and membranes pelleted by ultracentrifugation at 150,000 × *g* and 4°C for 1 h. Membrane preparations were washed, protein levels were determined by SDS-PAGE followed by Western blot with StrepTag II monoclonal antibodies (Novagen, used at 1:1,000 dilution from 200 µg/mL solution) and immunoglobulin G-alkaline phosphatase conjugates (at 1:3,000 dilution).

Western blots with antibodies detecting the GroEL chaperonin were performed as loading controls for soluble extracts and membrane extracts, respectively. GroEL antibodies were kindly provided by Dr. Alejandro Viale (IBR, CONICET-UNR, Argentina).

### Protein expression and purification

Soluble domains, i.e., the truncated soluble versions of CJO-1 (residues 17 to 248) and CIM-2 (residues 21 to 252), cloned as above into the pET28a-TeV vector, were overexpressed in *E. coli* BL21(DE3). The bacterial culture was grown at 37°C in LB medium supplemented with 50 µg/mL kanamycin until it reached OD_600_ 0.6-0.8. Then, protein expression was induced by the addition of 0.15 mM IPTG and 0.5 mM ZnSO_4_ to the medium. Cells were incubated overnight with shaking (220 rpm) at 18°C, pelleted and resuspended in lysis buffer (50 mM Tris-HCl pH 8.0, 200 mM NaCl, 10 mg/mL DNAse, 4 mM MgCl_2_, 2 mM PMSF, 10 mM β-mercaptoethanol). The cells were lysed by sonication, and the insoluble material was removed by centrifugation at 30,000 × *g* and 4°C for 1 h. After 30 min of starting centrifugation, 10 mg/mL streptomycin sulfate was added. The protein was purified using Ni-Sepharose affinity chromatography, the His-tag was cleaved by treatment with His_6_-tagged TEV protease (Sigma-Aldrich, manufacturer protocol), and the tag was removed by a second chromatographic step with the Ni-Sepharose resin in which the His_6_-tagged TEV protease was also retained. The purified protein was obtained in HEPES (pH 7.5) and 200 mM NaCl and concentrated using a 10 kDa molecular weight cut-off Centricon device (Millipore, Bedford, MA, USA). The protein concentration was measured spectrophotometrically using ɛ_280_ = 22920 M^−1^ cm^−1^ for CJO-1 and ɛ_280_ = 24410 M^−1^ cm^−1^ for CIM-2 (calculated using Expasy ProtParam [58], available at https://web.expasy.org/protparam/).

### Steady-state kinetics

β-Lactamase activity was followed in a JASCO V-670 spectrophotometer at 30°C in 10 mM HEPES (pH 7.5) and 200 mM NaCl supplemented with 15 µM ZnSO_4_ and 50 µg/mL bovine serum albumin. Substrates were used in the micromolar range, whereas the enzymes were used in the nanomolar range, to ensure pseudo-first-order kinetics. The initial reaction rates were calculated using the differential extinction coefficients for each β-lactam: piperacillin, Δɛ_235_ = −820 M^−1^ cm^−1^; imipenem, Δɛ_300_ = −9,000 M^−1^ cm^−1^; cefotaxime, Δɛ_260_ = −7,500 M^−1^ cm^−1^; cefepime, Δɛ_260_ = −10,000 M^−1^ cm^−1^; and ceftazidime, Δɛ_260_ = −9,000 M^−1^ cm^−1^; and the kinetic parameters *K_M_* and *k*_cat_ values determined by non-linear least squares fitting of their substrate concentration dependence to the Michaelis-Menten equation using GraphPad Prism 9 (GraphPad Software, San Diego, CA).

### Crystal structures of CJO-1 and CIM-2

CIM-2 and CJO-1 were crystallized by sitting drop vapor diffusion in MRC 96-well crystallization plates at 18°C. CIM-2 crystals formed by mixing 0.2 µL of protein (26 mg/mL) with 0.2 µL of crystallization reagent [0.1M Tris/Bicine pH8.5, 0.12 M (1,6-hexanediol, 1-butanol, 1,2-propanediol, 2-propanol, 1,4-butanediol, 1,3-propanediol), 37.5% (MPD, PEG1000, Peg3350)] from the Morpheus commercial screen (59) (Molecular Dimensions), and equilibrating against 50 µL reagent. Crystals grew to a maximum size in 1–2 months and were harvested for data collection by addition of 3 µL of crystallization reagent to the well before looping and flash-cooling in liquid nitrogen. CJO-1 crystals formed by mixing 0.2 µL of 29 mg/mL protein with 0.2 µL of crystallization reagent (0.2 M MgCl_2_, 0.1 M Bis-Tris pH 5.5, 25% [wt/vol] PEG 3350) from the Top96 commercial screen (Molecular Dimensions), and equilibrating against 50 µL reagent. For data collection, crystals were harvested and cryo-protected by brief exposure to crystallization reagent supplemented with 20% glycerol prior to flash-cooling in liquid nitrogen.

Diffraction data for CJO-1 and CIM-2 were collected at Diamond Light Source beamline I03 at the Zn-edge (wavelength = 1.27012/1.27021 Å, respectively), with structures determined using the SAD method. Data were processed using the xia2 pipeline (60), and integrated, scaled, and merged in autoPROC (61) and STARANISO(CJO-1) or in Dials (62) (CIM-2). Phases were solved for both, and initial models built, using the CRANK2 (63) pipeline in the CCP4 suite (64). CJO-1 crystallized in the *C*222_1_ space group (one molecule in the asymmetric unit [ASU]); two zinc ions were located (occupancies 1.00 and 0.69), with mean figure of merits (FOMs) before and after density modification of 0.2798/0.5574, respectively. The initial model was built with an R_work_/R_free_ of 0.2545/0.2681 and a FOM of 0.9148. CIM-2 crystallized in the *C*2 space group (two molecules in the ASU); four zinc ions were located with occupancies of 1.00/0.98/0.95/0.93, and a mean FOM before and after density modification of 0.1592/0.4449, respectively. The initial model was built and refined with an R_work_/R_free_ of 0.2571/0.2702 and a FOM of 0.8925. CIM-2 and CJO-1 structures were completed with iterative rounds of model building in Coot (65) and refinement in Phenix (66) and validated by Molprobity (67) and Phenix. Coordinates and structure factors have been deposited to the worldwide PDB under accession codes 9GX8 (CIM-2) and 9GX9 (CJO-1).

### Molecular dynamics simulations

For the initial structure of CJO-1, crystallographic data obtained in this work was used (PDB 9GX9). The initial structure for NDM-1 was obtained from the Protein Data Bank (PDB code 5ZGX [68]). Lys59Leu variant of CJO-1 was built *in silico* from the crystal structure of CJO-1.

All molecular dynamics simulations were performed using the Amber22 package (69). Each initial structure was prepared using Tleap and immersed in a truncated octahedral box of TIP3P water (70) with a 0.1 M concentration of NaCl. The ff14SB force field was used for the protein (71), with parameters for the active site consisting of zinc and hydroxide ions, and coordinated residues taken from literature (72). Each structure was first minimized, taken from a temperature of 0 K to 300 K at constant volume for 100 ps, then equilibrated at a constant pressure of 1 bar for 200 ps, before the final production time of 1 µs at constant pressure and temperature. For temperature and pressure control, the Langevin thermostat (73) and Monte Carlo barostat (74) were used.

Representative structures from each molecular dynamic simulation were obtained by clustering similar conformations based on RMSD and taking the most populated cluster (which in all cases represents more than 75% of the population). The clustering method used is the hierarchical agglomerative approach from the cpptraj module of Amber22. Electrostatic potential surfaces were calculated using APBS software (75). Figures showing surface visualization were produced using VMD software (76), with quicksurf drawing method with a radius scale of 0.8, a density isovalue of 0.9, and a color scale data range from −8 to 8 *k*_B_T/e_c_.

## SUPPLEMENTAL MATERIAL

Supplemental material aac.01918-24-s0001.docx Fig. S1 to S3; Tables S1 and S2; captions for Movies S1 and S2.

Movie S1 aac.01918-24-s0002.avi Animation of 1 µs of molecular dynamics simulation of CJO-1 in cartoon representation. Lys59, Phe64 and Tyr233 are shown in licorice, Zn2+ ions and the oxygen atom of the hydroxyl ion are shown as spheres. Hydrogen atoms are omitted for clarity.

Movie S2 aac.01918-24-s0003.avi Animation of 1 µs of molecular dynamics simulation of NDM-1 in cartoon representation. Leu59, Phe64 and Asn233 are shown in licorice, Zn2+ ions and the oxygen atom of the hydroxyl ion are shown as spheres. Hydrogen atoms are omitted for clarity.

ASM does not own the copyrights to Supplemental Material that may be linked to, or accessed through, an article. The authors have granted ASM a non-exclusive, world-wide license to publish the Supplemental Material files. Please contact the corresponding author directly for reuse.
