# Supplementary material for: Active site loops of membrane-anchored metallo-β-lactamases from environmental bacteria determine cephalosporinase activity
Source: Antimicrob Agents Chemother. 2025 Jun 23;69(8):e01918-24. doi: 10.1128/aac.01918-24 (PMC12326980; doi:10.1128/aac.01918-24)
Supplement: Supplemental material — Fig. S1 to S3; Tables S1 and S2; captions for Movies S1 and S2. [file aac.01918-24-s0001.docx]

**Supplementary information**

Figure S1. Phylogenetic tree of putative *Chryseobacterium* B1 metallo-beta-lactamases from the NCBI non-redundant protein database constructed with MEGA (Molecular evolutionary genetic analysis)*^4^*(Kumar et al., 2024); including CJO-1 (WP_076355270.1, green marked), CIM-2 (WP_062697499.1, orange marked) and NDM-1 (AVI24152.1, light blue marked). The percentage of replicate trees in which the associated taxa clustered together (500 replicates) is shown next to the branches.


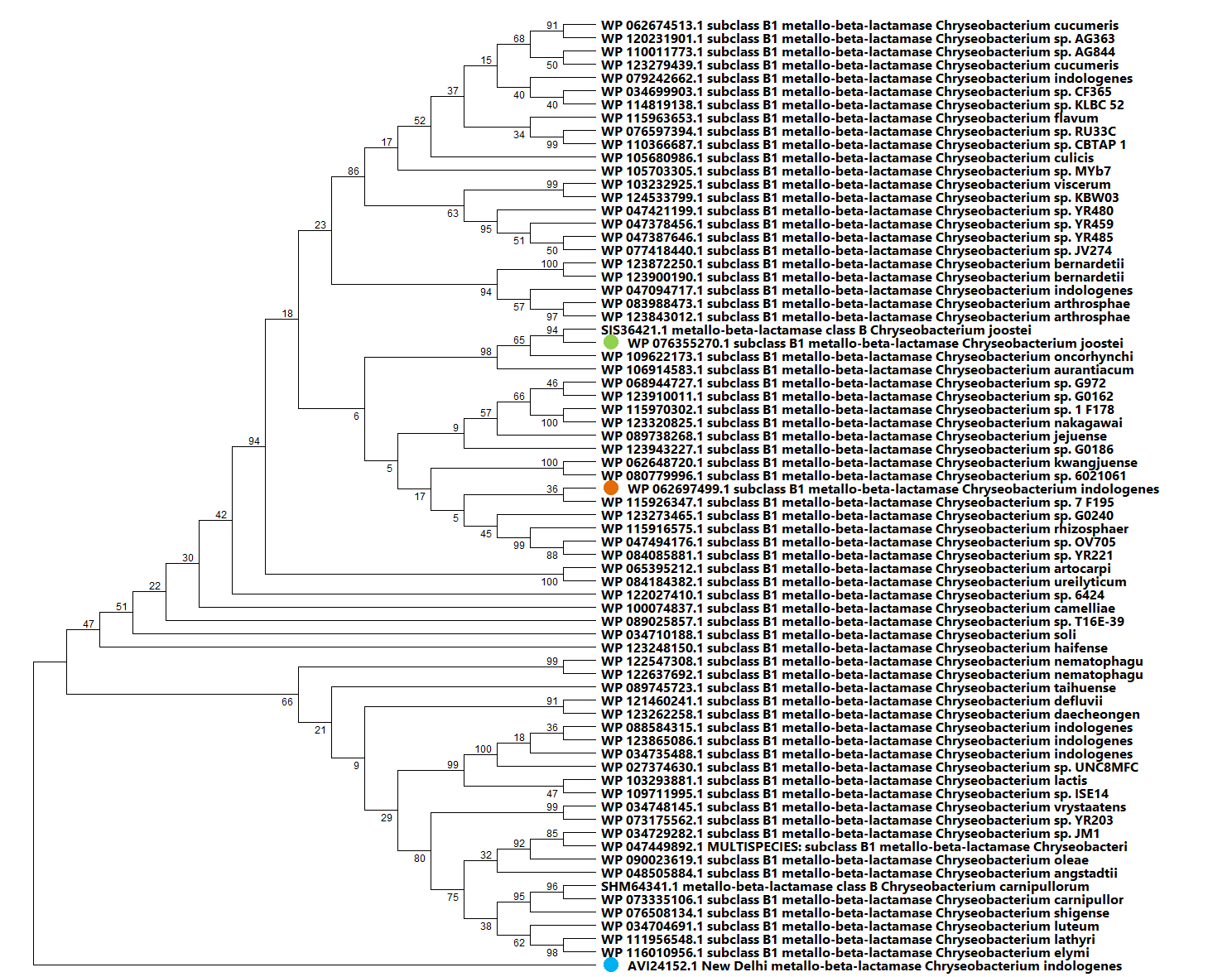

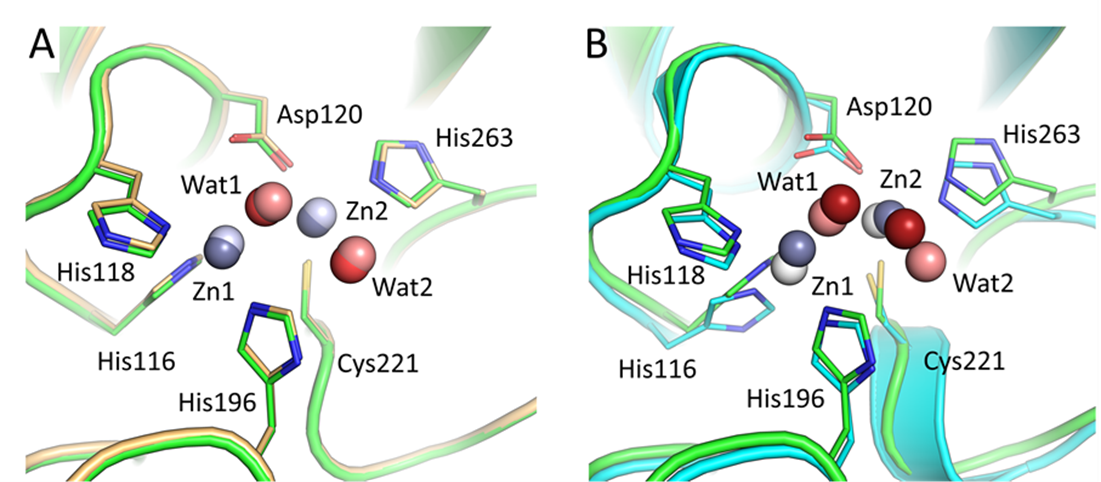


**Figure S2. Active site architectures of CJO-1, NDM-1 and CIM-2.** Views of the active sites of CJO-1 (carbon atoms green) with CIM-2 (carbon atoms orange) superimposed **(A)**. Zinc ions and water molecules are shown as gray and red spheres, respectively, with CJO-1 light colored. Superimposition of the active sites of CJO-1 and NDM-1 (carbon atoms cyan, PDB 5ZGY) **(B)**. For clarity, CIM-2 which is highly similar to CJO-1, is omitted. Zinc ions and water molecules are shown as gray and red spheres, respectively, with CJO-1 light colored and NDM-1 dark colored.


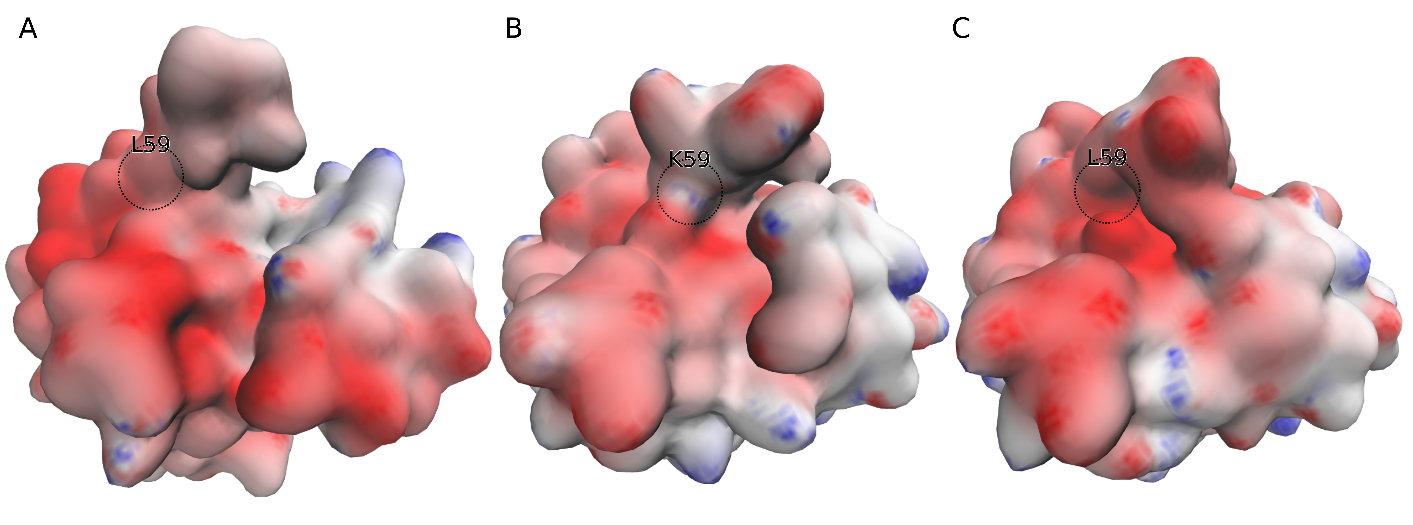


**Figure S3. Electrostatic potential surfaces of NDM-1 (A), CJO-1 (B) and CJO-1 K59L (C).**  Negative, neutral and positive potential are colored as red, white and blue, respectively, in a scale from -8 to 8 *k*_B_T/e_c_.

| **Table S1. X-ray crystallographic data collection and refinement statistics** | | |
| --- | --- | --- |
|  | **CJO-1** | **CIM-2** |
| **PDB accession** | 9GX9 | 9GX8 |
| **Data collection** |  |  |
| Space group | *C*222_1_ | *C*2 |
| Molecules/ASU | 1 | 2 |
| Cell dimensions |  |  |
| *a*, *b*, *c* (Å) | 47.52, 68.16, 135.00 | 164.29, 43.13, 75.69 |
| α, β, γ (°) | 90.0, 90.0, 90.0 | 90.0, 115.96, 90.0 |
| Wavelength (Å) | 1.27012 | 1.27021 |
| Resolution (Å) | 67.50 – 1.343 (1.46 – 1.34) | 73.86 – 1.56 (1.59 – 1.56) |
| *R*pim | 0.019 (0.356) | 0.044 (1.053) |
| CC ½ | 1.000 (0.719) | 0.998 (0.283) |
| *I* / σ*I* | 18.3 (1.5) | 12.0 (0.5) |
| Completeness (%) | 92.8  (51.9) | 99.2  (96.5) |
| Redundancy | 12.5 (6.8) | 6.7 (5.4) |
|  |  |  |
| **Refinement** |  |  |
| Resolution (Å) | 67.5 – 1.34 | 41.76 – 1.56 |
| No. reflections | 39330 | 67580 |
| *R*work / *R*free | 14.21 / 17.19 | 16.80 / 19.46 |
| No. non-H atoms |  |  |
| Protein | 1830 | 3642 |
| Solvent | 258 | 476 |
| Zinc ions | 2 | 4 |
| *B*-factors |  |  |
| Protein | 20.30 | 29.01 |
| Solvent | 32.65 | 38.77 |
| Zinc ions | 20.39 | 28.24 |
| R.m.s. deviations |  |  |
| Bond lengths (Å) | 0.009 | 0.006 |
| Bond angles (°) | 1.074 | 0.783 |
| Ramachandran (%) |  |  |
| Outliers | 0.44 | 0.45 |
| Favored | 96.44 | 96.62 |

| **Table S2. Zinc co-ordination distances in active sites** | | | | | | | | | |  |  |
| --- | --- | --- | --- | --- | --- | --- | --- | --- | --- | --- | --- |
|  |  | | **Distance (**Å**)** | | | | |  | | |  |
| **B1 MBL** | | **PDB code** | | **Zn1-Zn2** | **Zn1-Wat1** | **Zn2-Wat1** | **Zn2-Wat2** | | **ref** | | |
| **CJO-1** | | 9GX9 | | 3.41 | 1.90 | 2.04 | 2.28 | | this work | | |
| **CIM-2** chain A | | 9GX8 | | 3.35 | 1.88 | 2.11 | 2.27 | | this work | | |
| **CIM-2** chain B | | 9GX8 | | 3.37 | 1.88 | 2.09 | 2.26 | | this work | | |
| **NDM-1** | | 5ZGY | | 3.49 | 1.96 | 2.00 | 2.37 | | ^1^Zhang et al, 2018 | | |
| **IMP-1** chain A | | 5EV6 | | 3.42 | 2.43 | 1.87 | 2.27 | | ^2^Hinchliffe et al, 2016 | | |
| **VIM-2** chain B | | 4BZ3 | | 3.51 | 1.94 | 2.04 | 2.18 | | ^3^Brem et al, 2016 | | |

**Legends for Supplementary Movies**

**Supplementary Movie 1.** Animation of 1 µs of molecular dynamics simulation of CJO-1 in cartoon representation. Lys59, Phe64 and Tyr233 are shown in licorice, Zn2+ ions and the oxygen atom of the hydroxyl ion are shown as spheres. Hydrogen atoms are omitted for clarity.

**Supplementary Movie 2.** Animation of 1 µs of molecular dynamics simulation of NDM-1 in cartoon representation. Leu59, Phe64 and Asn233 are shown in licorice, Zn2+ ions and the oxygen atom of the hydroxyl ion are shown as spheres. Hydrogen atoms are omitted for clarity.

**References**

1. Zhang H, Ma G, Zhu Y, et al. Active-site conformational fluctuations promote the enzymatic activity of NDM-1. Antimicrob Agents Chemother. 2018;62(11). doi:10.1128/AAC.01579-18

2. Hinchliffe P, González MM, Mojica MF, et al. Cross-class metallo-β-lactamase inhibition by bisthiazolidines reveals multiple binding modes. *Proc Natl Acad Sci U S A*. 2016;113(26):E3745-E3754. doi:10.1073/pnas.1601368113

3. Brem J, Van Berkel SS, Zollman D, et al. Structural basis of metallo-β-lactamase inhibition by captopril stereoisomers. *Antimicrob Agents Chemother*. 2016;60(1):142-150. doi:10.1128/AAC.01335-15

4. Kumar S, Stecher G, Suleski M, Sanderford M, Sharma S, Tamura K. MEGA12: Molecular Evolutionary Genetic Analysis Version 12 for Adaptive and Green Computing. *Mol Biol Evol*. 2024;41(12). doi:10.1093/MOLBEV/MSAE263
